# Genetic evidence suggests a protective role of immunoglobulin M in Alzheimer's disease

**DOI:** 10.1002/alz.71865

**Published:** 2026-09-20

**Authors:** Shihui Peng, Guillaume Butler‐Laporte, Sterling C. Johnson, Corinne D. Engelman, Tianyuan Lu

**Affiliations:** ^1^ Department of Population Health Sciences School of Medicine and Public Health University of Wisconsin‐Madison Madison Wisconsin USA; ^2^ Department of Epidemiology Biostatistics and Occupational Health McGill University Montréal Quebec Canada; ^3^ Lady Davis Institute Jewish General Hospital McGill University Montréal Quebec Canada; ^4^ Centre for Human Genetics University of Oxford Oxford UK; ^5^ Division of Geriatrics and Gerontology Department of Medicine School of Medicine and Public Health University of Wisconsin‐Madison Madison Wisconsin USA; ^6^ Wisconsin Alzheimer's Disease Research Center Department of Medicine University of Wisconsin School of Medicine and Public Health Madison Wisconsin USA; ^7^ Wisconsin Alzheimer's Institute University of Wisconsin School of Medicine and Public Health Madison Wisconsin USA; ^8^ Department of Biostatistics and Medical Informatics School of Medicine and Public Health University of Wisconsin‐Madison Madison Wisconsin USA; ^9^ Center for Precision Medicine University of Wisconsin‐Madison Madison Wisconsin USA; ^10^ Center for Genomic Science Innovation University of Wisconsin‐Madison Madison Wisconsin USA; ^11^ Center for Demography of Health and Aging University of Wisconsin‐Madison Madison Wisconsin USA

**Keywords:** Alzheimer's disease, amyloid beta, immunoglobulin M, Mendelian randomization, plasma biomarkers, polygenic risk score, tau

## Abstract

**INTRODUCTION:**

Immune dysfunction has been implicated in Alzheimer's disease (AD), but the roles of specific immunoglobulin classes remain unclear.

**METHODS:**

We integrated human genetics and plasma biomarker analyses to evaluate immunoglobulin G (IgG), IgA, and IgM in relation to AD. Two‐sample Mendelian randomization analyses were conducted with multiple sensitivity analyses. Polygenic risk scores for immunoglobulin classes were developed in the All of Us Research Program and tested in the UK Biobank for associations with AD and dementia, and in two Wisconsin‐based cohorts for associations with plasma amyloid beta (Aβ)42/40, phosphorylated tau 217 (p‐tau217), neurofilament light chain (NfL), and glial fibrillary acidic protein (GFAP).

**RESULTS:**

Higher genetically proxied IgM was consistently associated with lower AD risk, higher Aβ42/40, and lower p‐tau217, but not with NfL or GFAP. No consistent associations were observed for IgG or IgA.

**DISCUSSION:**

IgM‐related humoral immunity may play a protective role in AD and warrants exploration for early intervention and prevention.

## Highlights

- Higher genetically proxied immunoglobulin M (IgM) is associated with lower Alzheimer's disease (AD) risk.
- Higher genetically proxied IgM is associated with more favorable amyloid and tau profiles.
- Genetically proxied IgG and IgA do not show consistent associations.

## 1 BACKGROUND

Alzheimer's disease (AD) represents a major and growing public health burden, with no curative treatment currently available. Therapeutic strategies targeting core pathological features such as amyloid beta (Aβ) and tau have shown limited and inconsistent clinical benefit, highlighting the need to identify additional biological mechanisms involved in disease pathogenesis.^1^, ^2^, ^3^, ^4^

Immunoglobulin‐based therapies have emerged as an important area of investigation in AD,^2^, ^5^ since immunoglobulins play important roles in humoral immunity through pathogen neutralization, immune regulation, and clearance of cellular debris and misfolded proteins.^6^, ^7^, ^8^, ^9^ The most studied immunoglobulin‐based therapy is intravenous immunoglobulin (IVIG), a mixture of polyclonal antibodies that contains most of the immunoglobulin G (IgG) found in the human immune repertoire.^10^ Earlier studies suggested that IVIG might bind Aβ and modulate AD‐related pathology, and its immune‐modulating and anti‐inflammatory effects represent other important mechanisms of action that may be relevant to AD treatment.^10^ Two small‐scale Phase 2 placebo‐control trials reported mixed results. Participants receiving IVIG treatments in certain treatment arms showed suggestive improvements in cognitive performance and cerebral metabolism, but there were no significant improvements in clinical outcomes.^11^, ^12^ A subsequent Phase 3 placebo‐controlled trial including 390 participants failed to demonstrate meaningful clinical benefits of IVIG over placebo in cognitive or functional outcomes, despite evidence of cerebrospinal fluid (CSF) penetration.^13^ Observational studies have reported inconsistent associations between circulating immunoglobulin levels and dementia‐related outcomes.^14^ One potential explanation for these inconsistent findings is that IVIG primarily reflects IgG‐mediated immune mechanisms,^15^ whereas other immunoglobulin classes, or broader aspects of humoral immunity, may be more relevant to AD risk and progression. Alternative approaches are therefore needed to clarify the roles of different immunoglobulin classes in AD.

Human genetic approaches offer a valuable opportunity to address this question. Because circulating immunoglobulin levels are influenced by environmental and clinical factors,^16^ analyses based on germline genetic variants may help clarify their relationships with AD risk, as these variants are randomly allocated at conception, fixed over the life course, and largely independent of environmental confounders.^17^ In this study, we aimed to systematically evaluate the relationship between three immunoglobulin classes (IgG, IgA, and IgM) and AD. First, we performed two‐sample Mendelian randomization (MR) analyses based on genome‐wide association studies (GWASs) to examine genetically informed associations between circulating immunoglobulin levels and AD risk, and CSF AD biomarkers, leveraging the largest available datasets to maximize statistical power. Second, we optimized polygenic risk scores (PRSs) in the National Institutes of Health (NIH) All of Us (AoU) Research Program and assessed whether genetic predisposition to each immunoglobulin was associated with AD and dementia outcomes in the UK Biobank, enabling evaluation of age‐dependent risk in a population‐based setting with multiple sensitivity analyses. Finally, we examined associations between genetically proxied immunoglobulin levels with AD‐specific plasma biomarkers, including Aβ42/40 ratio and phosphorylated tau 217 (p‐tau217), and neurodegeneration‐related plasma biomarkers, including neurofilament light chain (NfL) and glial fibrillary acidic protein (GFAP), in the Wisconsin Registry for Alzheimer's Prevention (WRAP) and Wisconsin Alzheimer's Disease Research Center (ADRC) cohorts. By integrating evidence across complementary approaches, we aimed to determine whether specific immunoglobulin classes are associated with AD risk and early AD‐related pathophysiology.

**RESEARCH IN CONTEXT**

1. **Systematic review**: Previous studies have suggested potential links between immunoglobulins and Alzheimer's disease (AD), but have presented inconsistent findings. These studies have largely emphasized overall immunoglobulin measures or IgG‐dominant mechanisms, without systematically evaluating differences across immunoglobulin classes.
2. **Interpretation**: Using complementary approaches, including Mendelian randomization, polygenic risk score analyses, and plasma biomarker evaluations across independent cohorts, we identified consistent evidence that higher genetically proxied IgM levels are associated with lower AD risk and more favorable amyloid and tau biomarker profiles. IgA and IgG showed no consistent associations. These findings support a potential protective role of IgM‐related humoral immunity in AD.
3. **Future directions**: Future work should investigate mechanisms linking IgM to amyloid and tau pathology and evaluate whether IgM‐mediated pathways can be targeted for early intervention or prevention. Extending these analyses to diverse populations and incorporating additional biomarkers will be important for validating and generalizing these findings.

## 2 METHODS

### 2.1 Mendelian randomization

GWASs for circulating immunoglobulin levels, AD, and CSF AD biomarkers are described in the Supplementary Notes. We performed two‐sample MR analyses to assess potential relationships between circulating immunoglobulin levels, AD outcomes, and CSF AD biomarkers. MR relies on three core assumptions^18^ ^:^ (1) relevance assumption—genetic instruments must be associated with the exposure of interest; (2) independence assumption—no confounders of the association between genetic instruments and the outcome; and (3) exclusion restriction assumption—genetic instruments are not related to the outcome other than via the exposure of interest (i.e., no horizontal pleiotropy). We retained genome‐wide significant variants (*p*‐value <5 × 10^−8^) for each immunoglobulin class based on the respective GWAS to fulfill the relevance assumption, and performed linkage disequilibrium (LD) clumping (r^2^ < 0.001, window = 1000 kb) based on a European ancestry reference panel from the 1000 Genomes Project (Phase 3).^19^

Notably, several selected variants are located in the major histocompatibility complex (MHC) region (GRCh38: chr6: 28,510,120–33,480,577). Because this region is highly pleiotropic and polymorphic,^20^ inclusion of variants within this region may introduce bias and complicate the interpretation of MR analyses. To address this, we conducted our primary analyses excluding variants in the MHC region. As a secondary analysis, we repeated the MR including MHC variants to assess the robustness of our findings and to evaluate whether signals from this immunologically relevant region influenced the results.

The independence and exclusion restriction assumptions cannot be verified from instrument selection alone and require additional sensitivity analyses.^18^, ^21^, ^22^ We conducted MR analyses separately using the clinically diagnosed AD GWAS,^23^ proxy AD GWAS,^24^ as well as GWAS of CSF Aβ42, CSF t‐tau, and CSF p‐tau181.^25^ The primary analysis was performed using the inverse‐variance weighted (IVW) random‐effects method,^26^ which provides the most precise estimates under the assumption that all genetic instruments are valid. To assess the robustness of our findings and examine potential violations of MR assumptions, we conducted sensitivity analyses using alternative methods, including the weighted median,^27^ penalized weighted median,^28^ weighted mode,^29^ and MR‐Egger methods.^27^ These approaches differ in their tolerance to invalid instruments and horizontal pleiotropy, thereby providing complementary evidence for inference. In particular, MR‐Egger regression was used to detect and account for directional horizontal pleiotropy through its intercept term. We also evaluated instrument strength by calculating F‐statistics for each genetic variant as well as for the full set of instruments. An F‐statistic greater than 10 was considered indicative of a low risk of weak instrument bias.^30^ All MR analyses were conducted using the TwoSampleMR R package (version 0.6.29).

To assess the possibility of reverse causation, we further performed reverse‐direction two‐sample MR analyses, using genetic liability to AD as the exposure^23^ and circulating immunoglobulin levels as the outcome.^31^ We used genome‐wide significant variants from the clinical AD GWAS as instruments and applied the same instrument selection criteria and analytical framework as in the primary MR analysis.

### 2.2 Association with AD and dementia

To complement our MR analyses, we constructed PRSs to evaluate the cumulative genetic contribution of immunoglobulin‐associated variants to AD risk at the individual level. We used the NIH AoU Research Program, a nationwide ongoing biomedical cohort across the United States,^32^ to optimize PRSs for IgG, IgA, and IgM. Details of PRS development are provided in the Supplementary Notes and Tables S1–S2.

We evaluated the associations between each optimized immunoglobulin PRS and AD outcomes in the UK Biobank, a large prospective population‐based cohort study comprising ≈500,000 participants ages 40–69 years recruited across the United Kingdom between 2006 and 2010.^33^ We retained 355,241 unrelated individuals of European ancestry to mitigate confounding due to population stratification. We defined two outcomes: (1) AD (International Classification of Diseases, 10th Revision (ICD‐10) codes: G30 or F00), and (2) dementia (ICD‐10 codes: G30, F00, F01, F02, or F03). Time‐to‐event was defined as the time from birth to first diagnosis of the outcome, death, or last follow‐up (Oct 31, 2022). Cox proportional hazard models were fitted, adjusting for apolipoprotein E (*APOE*) genotype, sex, genotyping batch, assessment center, and the first 10 genetic principal components. Proportional hazards assumptions were evaluated using Schoenfeld residuals test. Additional sensitivity analyses are described in the Supplementary Notes.

### 2.3 Association with plasma biomarkers

To examine whether the potential associations found in the UK Biobank were reflected in plasma biomarkers of AD pathology and neurodegeneration, we evaluated associations between immunoglobulin PRSs and plasma AD‐specific and neurodegenerative markers using data from the WRAP and Wisconsin ADRC cohorts, two longitudinal cohorts established in Wisconsin that together provide deeply phenotyped participants across the AD spectrum. Details of these two cohorts are provided in the Supplementary Notes.

We examined associations between immunoglobulin PRSs and plasma biomarkers using linear regression models in the combined WRAP/Wisconsin ADRC study population (*N* = 1413). Outcomes included plasma Aβ42/40 ratio as well as plasma p‐tau217, NfL, and GFAP levels. Plasma Aβ42, Aβ40, NfL, and GFAP levels were measured using the Simoa Human Neurology 4‐Plex E assay, and plasma p‐tau217 levels were measured using the Simoa pTau217 v2 assay on the Quanterix HD‐X platform. Plasma p‐tau217 measurements were standardized across instrument replacement and kit lot changes through crosswalk calibration using shared reference samples and linear regression to a common reference kit lot.^34^ After merging WRAP and Wisconsin ADRC cohorts, p‐tau217, NfL, and GFAP levels were log‐transformed prior to the analysis. All biomarker levels were then standardized to have zero mean and unit variance. Models were adjusted for *APOE* genotype, age at the latest measurement, age,^2^ sex, study cohort (WRAP or Wisconsin ADRC), and the first 10 genetic principal components.

For PRSs that showed significant association with any biomarkers, we further conducted stratified analyses by *APOE* ε4 carrier status to assess potential effect heterogeneity, where carriers were defined as individuals with at least one ε4 allele.

## 3 RESULTS

**FIGURE 1:**
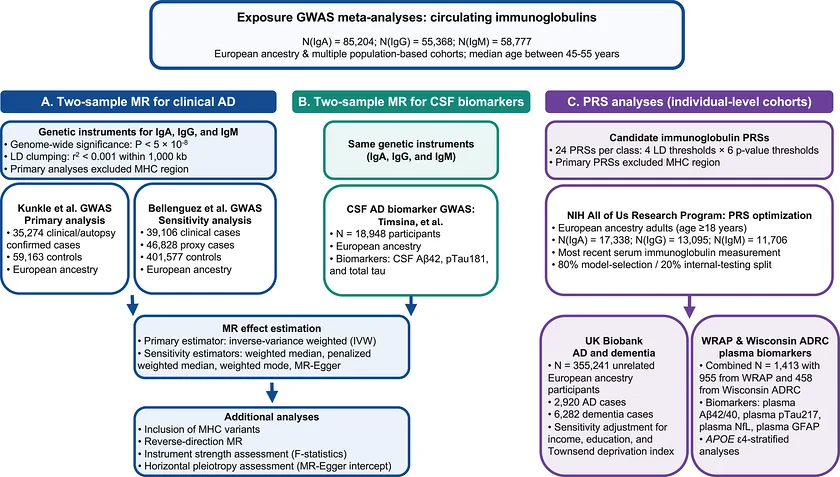
Overview of study design. We leveraged genome‐wide association study (GWAS) meta‐analyses for circulating immunoglobulin G (IgG), IgA, and IgM levels to conduct two‐sample Mendelian randomization (MR) and polygenic risk score (PRS) analyses. Primary MR analyses used an Alzheimer's disease (AD) GWAS of clinically diagnosed cases, whereas secondary analyses used a GWAS including both clinically diagnosed and proxy AD cases based on family history. MR estimates were obtained using five methods. Additional analyses included MR with major histocompatibility complex (MHC) variants included as instruments and reverse MR testing the association of AD genetic liability with immunoglobulin levels. In parallel, multiple candidate PRSs were constructed from GWAS summary statistics, and the optimal PRS for each immunoglobulin class was selected based on predictive performance in the National Institutes of Health (NIH) All of Us (AoU) Research Program. Associations between PRSs and AD and dementia were evaluated in the UK Biobank using Cox proportional hazards models, with sensitivity analyses using logistic regression and log‐rank tests with Kaplan–Meier curves. Additional sensitivity analyses adjusted for potential confounders. Associations between PRSs and plasma biomarkers, including amyloid beta (Aβ)42/40, phosphorylated tau 217 (p‐tau217), neurofilament light chain (NfL), and glial fibrillary acidic protein (GFAP) levels, were assessed in the Wisconsin Registry for Alzheimer's Prevention (WRAP) and Wisconsin Alzheimer's Disease Research Center (ADRC) cohorts, with additional analyses stratified by apolipoprotein E (*APOE*) ε4 status.

The study design is illustrated in Figure 1.

### 3.1 MR analyses

In the two‐sample MR analyses, instrument strength was adequate (F‐statistics > 10) (Tables S3–S6), and there was no evidence of directional horizontal pleiotropy (MR‐Egger intercept *P* > 0.05) (Table S7–S12).

**FIGURE 2:**
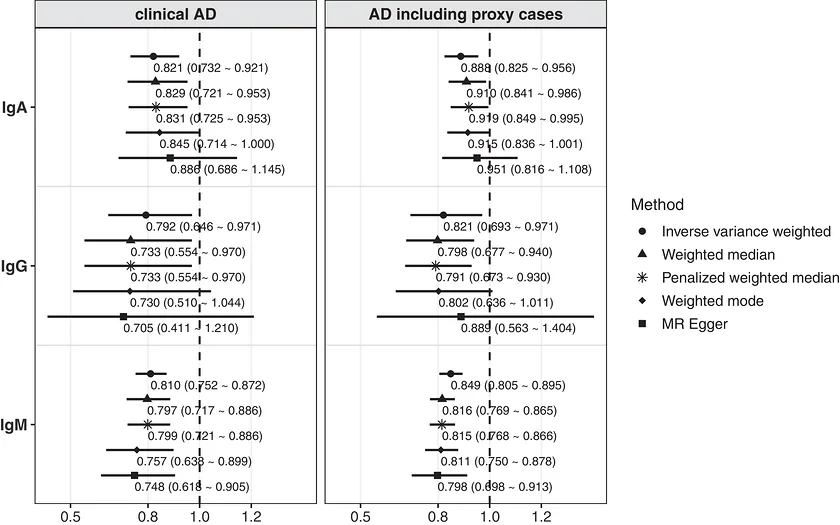
Mendelian randomization estimates of the association between genetically proxied immunoglobulin (Ig) levels and Alzheimer's disease (AD). Associations of genetically proxied IgA, IgG, and IgM levels with clinical AD and AD including proxy cases were evaluated using five Mendelian randomization methods. Dots represent odds ratios, with error bars indicating 95% confidence intervals. Different dot shapes denote results obtained using different Mendelian randomization methods. The dashed vertical line indicates the null.

Higher genetically proxied IgM levels were associated with lower AD risk in the primary analysis using clinically diagnosed AD (IVW odds ratio [OR] = 0.810 per one standard deviation [SD] increase, 95% confidence interval [CI]: 0.752–0.872, *p* = 2.6×10^−8^). Consistent effect direction and similar effect estimates were observed across all MR methods (Figure 2). IgA (IVW OR = 0.821, 95% CI: 0.732–0.921, *p* = 8.1 × 10^−4^) and IgG (IVW OR = 0.792, 95% CI: 0.646–0.971, *p* = 2.5 × 10^−2^) also showed suggestive associations, although estimates from alternative MR methods were not consistently statistically significant despite a similar direction of effect (Figure 2). Inclusion of MHC variants yielded highly consistent trends across all analyses (Table S7). Using the proxy‐inclusive AD GWAS, similar results were observed with or without inclusion of MHC variants (Table S8). Replication analyses using independent FinnGen GWAS^35^ of circulating Ig levels yielded highly consistent evidence for the association between genetically proxied IgM levels and lower AD risk, whereas associations for IgA and IgG were not consistently observed across MR methods (Supplementary Notes and Tables S9–S10).

Furthermore, higher genetically proxied IgM levels were associated with higher CSF Aβ42 levels (IVW *β* = 0.118 per one SD increase, 95% CI: 0.064–0.172, *p* = 1.9×10^−5^), lower CSF p‐tau181 levels (IVW *β* = −0.071, 95% CI: −0.130 to −0.013, *p* = 1.7×10^−2^) and lower CSF t‐tau levels (IVW *β* = −0.079, 95% CI: −0.134 to −0.024, *p* = 4.8×10^−3^). Directionally consistent associations were also observed using alternative MR methods, although statistical significance was reduced, likely because of limited sample sizes of the CSF biomarker GWAS. Associations between IgG and CSF AD biomarkers were less prominent, whereas IgA showed no significant associations. Inclusion of MHC variants yielded similar overall patterns across analyses (Table S11). Reverse MR analyses did not provide evidence that genetic liability to AD influences immunoglobulin levels, with no consistent associations observed after multiple testing correction (Table S12).

### 3.2 Association of immunoglobulin PRSs with AD and dementia in UK Biobank

A total of 355,241 unrelated European ancestry participants from the UK Biobank were included in the PRS association analyses, including 2920 clinically diagnosed AD cases (0.8%) and 6282 dementia cases (1.8%) (Table S13). Among AD cases, 50.9% were female and the mean age at diagnosis was 75.2 years. Among dementia cases, 46.3% cases were female, and the mean age at diagnosis was 74.8 years.

**FIGURE 3:**
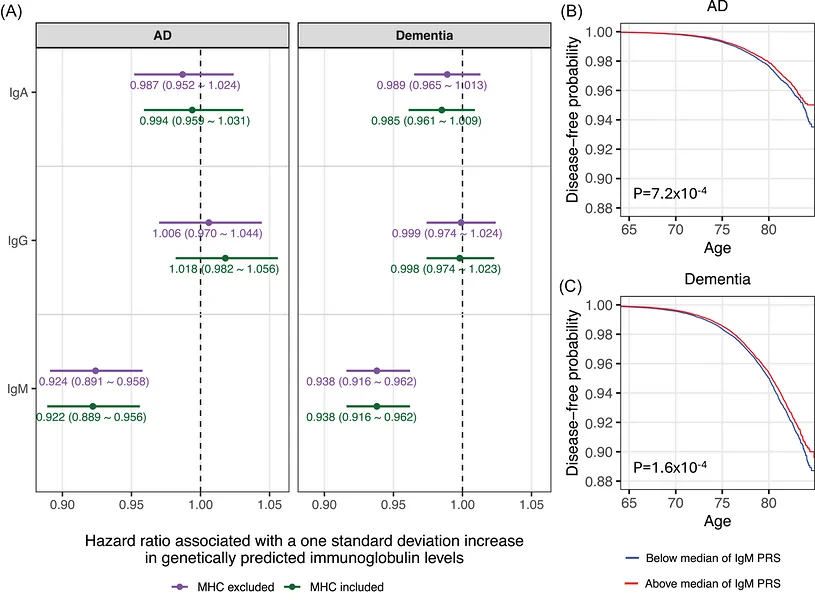
Association between genetically proxied immunoglobulin levels and Alzheimer's disease (AD) outcomes in the UK Biobank. (A) Cox proportional hazards models were used to estimate hazard ratios for AD and dementia associated with a 1 standard deviation increase in genetically proxied IgA, IgG, and IgM levels. Dots represent hazard ratio estimates, and error bars indicate 95% confidence intervals. Results from primary analyses excluding major histocompatibility complex (MHC) variants and sensitivity analyses including MHC variants were illustrated. Dashed vertical lines denote the null. Kaplan–Meier curves display disease‐free probability for (B) AD and (C) dementia. Blue curves represent participants with an immunoglobulin M (IgM) polygenic risk score (PRS) below the median, and red curves represent participants with an IgM PRS above the median. Participants were censored at age 85 years due to limited sample size beyond this age. Log‐rank test *p*‐values comparing these two groups are provided.

In the primary analyses, each SD increase in IgM PRS was associated with a lower hazard of AD (hazard ratio [HR] = 0.924, 95% CI: 0.891–0.958, *p* = 2.0×10^−5^) and dementia (HR = 0.938, 95% CI: 0.916–0.962, *p* = 4.8×10^−7^). Results were highly consistent when the PRS included MHC variants. In contrast, there was no evidence of association for either IgA or IgG PRS with either outcome (Figure 3AB, Table S14). There was no evidence of violation of proportional hazards assumption (Table S15).

Additional sensitivity analyses showed that the associations between IgM PRS and both AD and dementia remained consistent after adjusting for Townsend index (AD: HR = 0.923, 95% CI: 0.890–0.957, *P* = 1.5×10^−5^; dementia: HR = 0.937, 95% CI: 0.914–0.961, *P* = 3.0×10^−7^), annual household income (AD: HR = 0.926, 95% CI: 0.888–0.966, *p* = 3.1×10^−4^; dementia: HR = 0.943, 95% CI: 0.917–0.970, *p* = 4.7×10^−5^), and years of education (AD: HR = 0.920, 95% CI: 0.886–0.954, *p* = 7.4×10^−6^; dementia: HR = 0.936, 95% CI: 0.913–0.960, *p* = 2.6×10^−7^), respectively (Table S16).

Kaplan–Meier curves further supported the IgM findings. Individuals with above‐median IgM PRS showed significantly lower cumulative incidence of AD and dementia compared to those below the median (log‐rank *p* = 7.2×10^−4^ for AD and *p* = 1.6×10^−4^ for dementia), with separation between survival curves becoming more apparent at older ages (Figure 3B,C). In secondary analyses using logistic regression excluding controls  <65 years of age, results were also consistent (Table S17). Each SD increase in IgM PRS was associated with lower odds of AD (OR = 0.929, 95% CI: 0.85–0.963, *p* = 7.5×10^−5^) and dementia (OR = 0.942, 95% CI: 0.919–0.966, *p* = 3.7×10^−6^), whereas IgA and IgG PRSs showed no evidence of association.

### 3.3 Association with plasma biomarkers in WRAP and Wisconsin ADRC cohorts

A total of 1413 unrelated European ancestry participants from the WRAP and Wisconsin ADRC cohorts were included in the biomarker analyses (Table 1). Overall, 64.9% of the participants were female. The mean age at latest biomarker measurement was 69.2 years for plasma Aβ42/40 ratio, NfL levels, and GFAP levels, and 70.0 years for plasma p‐tau217 levels.

**TABLE 1:** WRAP and Wisconsin ADRC cohort characteristics.

	Wisconsin ADRC^a^	WRAP^b^	Combined
**Sample size**	458	955	1413
**Female, *N* (%)**	261 (57.0)	656 (68.7)	917 (64.9)
**Education years, mean (SD)**	16.2 (2.7)	16.0 (2.2)	16.0 (2.4)
**Body mass index, median (Q1, Q3)**	27.1 (23.7, 30.2)	27.5 (24.3, 31.9)	27.4 (24.1, 31.3)
***APOE* genotype, *N* (%)**			
ε**2/**ε**2**	2 (0.4)	4 (0.4)	6 (0.4)
ε**2/**ε**3**	47 (10.3)	79 (8.3)	126 (8.9)
ε**3/**ε**3**	210 (45.9)	522 (54.7)	732 (51.8)
ε**2/**ε**4**	18 (3.9)	23 (2.4)	41 (2.9)
ε**3/**ε**4**	137 (29.9)	300 (31.4)	437 (30.9)
ε**4/**ε**4**	44 (9.6)	27 (2.8)	71 (5)
**Aβ42/40 ratio, median (Q1, Q3)**	0.067 (0.059, 0.078)	0.066 (0.058, 0.075)	0.067 (0.058, 0.075)
**NfL, pg/mL, median (Q1, Q3)**	18.0 (13.1, 23.5)	17.5 (13.3, 24.4)	17.6 (13.2, 24.2)
**GFAP, pg/mL, median (Q1, Q3)**	119.7 (89.1, 186.6)	127.9 (90.0, 175.3)	126.3 (89.7, 176.0)
**p‐tau217, pg/mL, median (Q1, Q3)**	0.359 (0.245, 0.602)	0.354 (0.252, 0.584)	0.355 (0.252, 0.590)
**Age at latest Aβ/NfL/GFAP measurement, mean (SD)**	69.3 (8.4)	69.2 (7.1)	69.2 (7.4)
**Age at latest p‐tau217 measurement, mean (SD)**	70.4 (8.1)	69.8 (7.2)	70.0 (7.4)

^a^ Wisconsin Alzheimer's Disease Research Center Clinical Core cohort.

^b^ Wisconsin Registry for Alzheimer's Prevention cohort.

**FIGURE 4:**
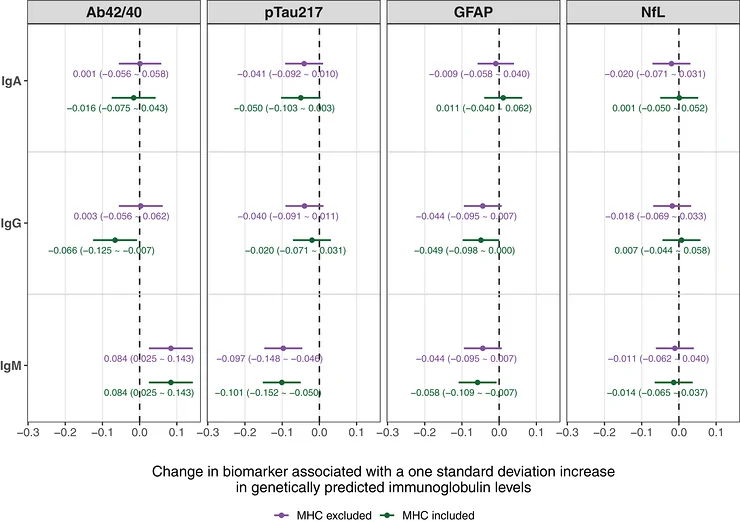
Association between immunoglobulin polygenic risk scores (PRSs) and plasma biomarkers in Wisconsin Registry for Alzheimer's Prevention (WRAP) and Wisconsin Alzheimer's Disease Research Center (ADRC) cohorts. Linear regression was used to estimate changes in plasma amyloid beta (Aβ)42/40, phosphorylated tau 217 (p‐tau217), glial fibrillary acidic protein (GFAP), and neurofilament light chain (NfL) levels associated with a 1 standard deviation increase in genetically proxied immunoglobulin A (IgA), IgG, and IgM levels. Dots represent effect estimates, and error bars indicate 95% confidence intervals. Colors indicate whether PRSs included major histocompatibility (MHC) variants. Dashed vertical lines denote the null.

In the primary analyses, each SD increase in IgM PRS was associated with higher plasma Aβ42/40 ratio ($\hat{\beta }$ = 0.084, 95% CI: 0.025–0.143, *p* = 5.0×10^−3^) and lower plasma p‐tau217 levels ($\hat{\beta }$ = 0.097, 95% CI: 0.046–0.148, *p* = 2.3×10^−4^). These associations were consistent when the PRS included MHC variants. The IgM PRS showed suggestive association with plasma GFAP levels when MHC variants were included ($\hat{\beta }$ = −0.058, 95% CI: −0.109 to −0.007, *p* = 0.025), but this suggestive association would not withstand multiple testing correction. No association was observed between IgM PRS and plasma NfL levels. In contrast, neither IgA nor IgG PRS showed consistent associations with any of the investigated biomarkers (Figure 4, Table S18).

In stratified analyses by *APOE* ε4 carrier status, associations between IgM and biomarkers were stronger among ε4 carriers (Table S19). Each SD increase in IgM PRS was associated with a 0.121 SD increase (95% CI: 0.029–0.213, *p* = 9.8×10^−3^) in Aβ42/40 ratio and a 0.163 SD decrease (95% CI: 0.069–0.257, *p* = 8.1×10^−4^) in p‐tau217 among ε4 carriers, compared to a 0.066 SD increase (95% CI: –0.010 to 0.142, *p* = 0.088) in Aβ42/40 ratio and a 0.059 SD decrease (95% CI: –0.002 to 0.120, *p* = 0.055) in p‐tau217 among non‐carriers. Results were similar when MHC variants were included.

## 4 DISCUSSION

In this study, we found consistent evidence that genetically proxied higher IgM levels are associated with lower risk of AD. This association remained robust across multiple sensitivity analyses using two AD GWAS designs and CSF AD biomarkers in MR analyses, was replicated in PRS‐based association analyses in the UK Biobank, and was further supported by associations with AD‐related biomarkers in WRAP and Wisconsin ADRC cohorts. In contrast, IgA and IgG showed no consistent or significant associations across analytical approaches, suggesting a more limited or indirect role in AD pathogenesis.

IgM plays a central role in innate‐like humoral immunity and can facilitate clearance of misfolded proteins, including Aβ.^7^, ^36^, ^37^, ^38^ Naturally occurring anti‐Aβ antibodies (NAbs‐Aβ), which are IgM predominant, have been associated with more favorable amyloid‐related and cognitive outcomes in prior studies.^39^, ^40^, ^41^ Because IgM is pentameric, it may be particularly effective at binding oligomeric Aβ assemblies,^42^, ^43^ potentially influencing early amyloid‐related processes upstream of tau pathology. Consistent with this, genetically proxied higher IgM levels were associated with lower AD risk and more favorable AD‐related biomarkers, including higher plasma Aβ42/40 ratio, lower plasma p‐tau217 levels, higher CSF Aβ42 levels, and lower CSF p‐tau181 and total tau levels. These biomarker patterns are consistent with reduced amyloid deposition and downstream tau‐related pathology.^40^, ^44^, ^45^

We did not observe significant associations between IgM PRS and plasma NfL or GFAP. This may reflect that these biomarkers capture more general or later‐stage neurodegeneration rather than AD‐specific pathology, whereas IgM may primarily influence AD‐specific pathways or earlier stages of disease progression, prior to substantial neurodegenerative change. However, it should be noted that the participants in WRAP and Wisconsin ADRC cohorts are relatively younger and might not yet exhibit substantial changes in GFAP and NfL, which could limit power to detect associations with these biomarkers. We further observed that the associations between IgM PRS and AD‐related biomarkers were stronger among *APOE* ε4 carriers. This suggests that IgM‐related pathways may be particularly relevant in individuals at high genetic risk due to *APOE* genotype. However, larger studies are needed to confirm these findings.

Our findings highlight differences across immunoglobulin classes. In contrast to IgM, we observed no consistent associations for IgG despite prior interest in IgG‐based therapies. One possible explanation is that IgG reflects a class‐switched adaptive immune response that may occur later in disease progression, after substantial amyloid deposition or neurodegeneration has developed.^2^, ^9^, ^46^ In addition, IgG‐mediated responses may contribute to neuroinflammation through Fc receptor–mediated microglial activation.^45^, ^47^, ^48^, ^49^ By contrast, IgM may reflect more upstream and sustained immune processes relevant to early disease pathogenesis. Differences in antigen specificity may also contribute. Specifically, total circulating IgG represents a heterogeneous antibody pool in which only a small fraction may be Aβ specific,^50^, ^51^, ^52^, ^53^, ^54^ whereas IgM is generally more polyreactive and involved in constitutive immune surveillance,^55^ potentially contributing to the more robust associations observed in our analyses. We also observed no consistent associations for IgA. This is biologically plausible because IgA primarily mediates mucosal and immunoregulatory functions rather than direct protein clearance pathways,^6^, ^56^ and prior observational studies have similarly reported inconsistent associations with dementia‐related biomarkers.^14^, ^57^

Our study has important limitations. First, our analyses were restricted to individuals of European ancestry, as non‐European ancestry data resources remain limited. Expanding analyses to diverse populations should be a priority for future work to assess the generalizability of our findings. Second, the variance explained by PRSs for immunoglobulin levels was modest, particularly for IgG. However, the overall pattern of results aligned with the null or inconsistent associations reported in existing observational studies and clinical trials. Third, our biomarker analyses relied on plasma‐based measurements, which are widely used, minimally invasive, and increasingly validated as informative peripheral indicators of neurodegenerative processes. Nevertheless, they may not fully capture central nervous system pathology or the burden of cerebral amyloid and tau deposition. Although our MR analyses on CSF biomarkers can strengthen our finding, future studies incorporating amyloid‐ and tau‐targeted positron emission tomography (PET) scans will still be important to further evaluate these associations. Fourth, the lack of association between IgM PRS and GFAP and NfL may reflect limited statistical power in a relatively younger study population, and future studies in older cohorts with greater neurodegenerative burden are warranted. Finally, our analyses rely on the implicit MR assumption that genetic instruments exert relatively stable effects on circulating immunoglobulin levels throughout the life course. Because the exposure GWAS combined multiple cohorts and only summary‐level data were available, we could not directly assess this assumption. Although germline genetic variants are fixed at conception, their effects on immunoglobulin levels may vary across different stages of life. Future age‐stratified or longitudinal GWAS of immunoglobulin levels will help evaluate whether MR findings are influenced by potential age‐dependent genetic effects.

In conclusion, our findings provide strong human genetic evidence that higher IgM levels may play a protective role in AD. These results highlight the importance of innate‐like humoral immunity in AD pathogenesis and suggest that IgM‐related pathways may warrant exploration for early intervention and prevention strategies.

## CONFLICT OF INTEREST STATEMENT

Tianyuan Lu has been providing consulting services to Five Prime Sciences Inc. for research programs unrelated to this work. Tianyuan Lu's spouse is an employee of Regeneron Pharmaceuticals Inc. Sterling C. Johnson has served as a consultant to Roche Diagnostics, Merck, and Prothena and has received research funding from Cerveau Technologies. The other authors have nothing to disclose. Author disclosures are available in the Supporting Information.

## CONSENT STATEMENT

All human subjects provided written informed consent. Access to the All of Us Research Program is governed by the program's data access policies and is available to registered researchers in accordance with those guidelines. The UK Biobank, Wisconsin Registry for Alzheimer's Prevention (WRAP), and Wisconsin Alzheimer's Disease Research Center (ADRC) data used in this study are available upon approval by the respective research committees.

## Supporting information




Supporting Information

Supporting Information

Supporting Information

Supporting Information
