# Supplementary material for: Genetic evidence suggests a protective role of immunoglobulin M in Alzheimer's disease
Source: Alzheimers Dement. 2026 Sep 20;22(9):e71865. doi: 10.1002/alz.71865 (PMC13590935; doi:10.1002/alz.71865)
Supplement: Supplementary file 3 [file ALZ-22-e71865-s004.docx]

## Genome-wide association studies for immunoglobulin classes

GWAS summary statistics for circulating immunoglobulin levels (IgA, IgG, and IgM) were obtained from the largest GWAS meta-analyses to date, including 85,204 individuals for IgA, 55,368 for IgG, and 58,777 for IgM, of European ancestry^1^. The GWAS summary statistics were downloaded from the corresponding Zenodo repository^2^. Across the contributing cohorts, the median age ranged from 45 to 55 years^3–7^. Immunoglobulin levels were measured using cohort-specific platforms, including immunosorbent assays and affinity-based proteomic assays such as SomaScan. Phenotypes were transformed and standardized within each contributing cohort using study-specific procedures, and summary statistics were combined using fixed-effect inverse-variance weighted meta-analysis after harmonization and rescaling across studies. Details of these meta-analyses have been described previously^1^.

**Genome-wide association studies for immunoglobulin classes from FinnGen**

To evaluate the robustness of our findings, we additionally obtained GWAS summary statistics for circulating immunoglobulin levels (IgA, IgG, and IgM) from FinnGen (Data Freeze 13), including 50,099 individuals for IgA, 27,675 for IgG, and 21,438 for IgM, all of Finnish ancestry. FinnGen is a nationwide research project integrating genotype data from Finnish biobank participants with longitudinal nationwide health registry data, including laboratory measurements obtained through the Finnish Kanta health records^8^. The median age at sample collection was 53 years. Laboratory measurements underwent quality control and phenotype-specific preprocessing before genome-wide association analyses, following the FinnGen analytical pipeline described at <https://www.finngen.fi/en/cohort_and_data>.

## Genome-wide association studies for AD

GWAS summary statistics for AD outcomes were obtained from two large GWAS meta-analyses. Our primary analysis used summary statistics from a clinically diagnosed late-onset AD GWAS conducted by Kunkle et al., comprising 35,274 clinical or autopsy-documented cases and 59,163 controls of European ancestry^9^. Each contributing cohort performed association analyses separately under additive genetic models, generally adjusting for age, sex, and principal components, with cohort-specific covariates included as needed. Summary statistics were generated from cohort-level GWAS imputed to the 1000 Genomes reference panel and meta-analyzed across participating studies. As a secondary sensitivity analysis, we used summary statistics from a larger GWAS conducted by Bellenguez et al. This predominantly European ancestral dataset included 39,106 clinically diagnosed AD cases, 46,828 proxy AD/dementia cases, and 401,577 controls^10^. Each contributing cohort conducted GWAS separately using study-specific models adjusted for age, sex, and principal components, with additional cohort-level covariates such as genotyping batch, recruitment site, or array platform included where appropriate before meta-analysis. We did not use the larger GWAS for primary analysis because recent evidence has shown that proxy AD GWAS are susceptible to substantial bias due to uncorrected survival bias and nonrandom participation in parental illness surveys, which can distort genetic correlations and downstream MR estimates^11^.

## Genome-wide association studies for CSF AD biomarkers

GWAS summary statistics for CSF AD biomarkers (Aβ42, t-tau, and pTau181) were obtained from the largest and latest GWAS meta-analyses, including up to 18,948 individuals of European ancestry from 30 studies^12^. Biomarker measurements were generated using cohort-specific CSF assays and harmonized within study prior to association analyses. GWAS were conducted separately within cohorts using standardized biomarker measures, followed by inverse-variance weighted meta-analyses across studies.

## Development of polygenic risk scores

To complement our MR analyses, we constructed PRSs for IgG, IgA, and IgM to evaluate the cumulative genetic contribution of immunoglobulin-associated variants to AD risk at the individual level. We generated multiple candidate PRSs using a clumping and thresholding approach with varying r^2^ thresholds (0.01, 0.05, 0.1, 0.5) and p-value thresholds (5x10^-3^, 5x10^-4^, 5x10^-5^, 5x10^-6^, 5x10^-7^, 5x10^-8^), resulting in 4x6 candidate PRSs for each immunoglobulin class. SNPs were filtered based on minor allele frequency > 0.01, and LD pruning was performed using a European ancestry reference panel from the 1000 Genomes Project (phase 3).

We used the NIH AoU Research Program, a nationwide, ongoing biomedical cohort designed to enroll over one million participants from diverse populations across the United States^13^, to obtain the optimized PRS for IgG, IgA, and IgM, respectively. There is no known sample overlap between the AoU and the GWAS populations. Serum immunoglobulin laboratory values in AoU were obtained from harmonized electronic health record laboratory data. Among 414,830 individuals with available short-read whole genome sequencing data, we included 17,338 European ancestry participants with available circulating IgA measurements, 13,095 with IgG measurements, and 11,706 with IgM measurements, all reported in mg/dL. Participants were excluded if the measurement values were outliers (defined as more than 2x the interquartile range below the first quartile or above the third quartile), if they were not part of a maximally unrelated set (kinship < 0.1, as provided by AoU^14^), or if they had low-quality genomic data (e.g., sample swaps, contamination, or preparation issues identified by AoU^14^). For each participant, we used the most recent immunoglobulin measurement. Participants younger than 18 years at their most recent measurement were also excluded.

We randomly split the dataset into a model selection dataset (80%) and a testing dataset (20%) with highly similar demographic characteristics (**Suppl. Table 1**). For each immunoglobulin class, we regressed measured immunoglobulin levels on each candidate PRS in the model selection dataset, adjusting for age upon the most recent immunoglobulin measurement, sex, and the first ten genetic principal components. We calculated the proportion of variance explained (R^2^) by each PRS and selected the best-performing PRS for each immunoglobulin based on this metric. We then evaluated the selected PRSs in the testing dataset using the same regression model.

Similar to the MR analyses, we constructed PRSs both with and without variants in the MHC region. For the downstream analyses, PRSs excluding MHC variants were used for the primary analysis and PRSs including MHC variants were used for sensitivity analysis.

Detailed performance metrics are available in **Suppl. Table 2**.

## UK Biobank sensitivity analyses

To assess whether the associations between immunoglobulin PRSs and AD outcomes would be consistent after removing the variances which were attributed to socioeconomic status, we conducted additional sensitivity analyses using Cox models by further adjusting for Townsend index, annual household income, and years of education, respectively. Years of education were derived from self-reported education qualifications and converted into years according to the International Standard Classification of Education (ISCED)^15^.

For PRSs that showed significant association with AD outcomes in the primary analysis, we generated Kaplan-Meier survival curves and compared cumulative incidence across PRS strata, dichotomized at the median into below-median and above-median groups, using log-rank test.

We also conducted logistic regression analyses as a secondary analysis to assess robustness of our findings. In these analyses, we restricted controls to individuals aged ≥ 65 years, acknowledging the potential for outcome misclassification among younger individuals who may not yet have reached the age of risk for AD. Models included the same covariates as in the primary analysis, with additional adjustment for age and age^2^. Age was defined as age at the first diagnosis for cases, age at death for deceased controls, or age at last follow-up (Oct 31^st^, 2022) for living controls.

**WRAP and Wisconsin ADRC cohorts**

WRAP is a risk-enriched observational cohort that has enrolled more than 1,700 cognitively unimpaired adults in midlife since 2001, with 73% of participants having a parental history of AD dementia^16^. The Wisconsin ADRC has enrolled over 1,100 participants aged 45 years and older with clinical classifications of cognitively unimpaired status, mild cognitive impairment, or dementia due to AD and/or non‐AD pathology since 2009^17^. All subjects provided signed informed consent before participation. This study was approved by the University of Wisconsin-Madison Institutional Review Board and was conducted in accordance with the Declaration of Helsinki.

We included unrelated individuals of European ancestry from the WRAP and the Wisconsin ADRC cohorts. Genetic ancestry was assigned using ADMIXTURE-based ancestry proportion estimates^18^ derived from comparison with the 1000 Genomes reference superpopulations^19^, and participants were classified as European ancestry if their inferred European ancestry proportion exceeded 90%. Relatedness was determined using genotype-based kinship inference (KING/PC-Relate pipeline^20^), and all related individuals identified through kinship estimation were excluded.

## Reference

1.	Willis, T. W. *et al.* Large-scale GWAS meta-analysis of serum antibody levels in healthy individuals reveals distinct genetic architectures. Preprint at https://doi.org/10.1101/2025.10.25.25338738 (2025).

2.	Willis, T. Data for the manuscript ‘Large-scale GWAS meta-analysis of serum antibody levels reveals distinct genetic architectures’. https://doi.org/10.5281/zenodo.17010403 (2025) doi:10.5281/zenodo.17010403.

3.	Dennis, J. K. *et al.* Clinical laboratory test-wide association scan of polygenic scores identifies biomarkers of complex disease. *Genome Med.* **13**, 6 (2021).

4.	Scepanovic, P. *et al.* Human genetic variants and age are the strongest predictors of humoral immune responses to common pathogens and vaccines. *Genome Med.* **10**, 59 (2018).

5.	Liu, L. *et al.* Genetic regulation of serum IgA levels and susceptibility to common immune, infectious, kidney, and cardio-metabolic traits. *Nat. Commun.* **13**, 6859 (2022).

6.	Pietzner, M. *et al.* Mapping the proteo-genomic convergence of human diseases. *Science* **374**, eabj1541 (2021).

7.	Ferkingstad, E. *et al.* Large-scale integration of the plasma proteome with genetics and disease. *Nat. Genet.* **53**, 1712–1721 (2021).

8.	Kurki, M. I. *et al.* FinnGen provides genetic insights from a well-phenotyped isolated population. *Nature* **613**, 508–518 (2023).

9.	Kunkle, B. W. *et al.* Genetic meta-analysis of diagnosed Alzheimer’s disease identifies new risk loci and implicates Aβ, tau, immunity and lipid processing. *Nat. Genet.* **51**, 414–430 (2019).

10.	Bellenguez, C. *et al.* New insights into the genetic etiology of Alzheimer’s disease and related dementias. *Nat. Genet.* **54**, 412–436 (2022).

11.	Wu, Y. *et al.* Pervasive biases in proxy genome-wide association studies based on parental history of Alzheimer’s disease. *Nat. Genet.* **56**, 2696–2703 (2024).

12.	Timsina, J. *et al.* GWAS meta-analysis of cerebrospinal fluid Alzheimer’s biomarkers reveals loci regulating lipids, brain volume and autophagy. *Nat. Commun.* https://doi.org/10.1038/s41467-026-71682-8 (2026) doi:10.1038/s41467-026-71682-8.

13.	The All of Us Research Program Investigators. The “All of Us” Research Program. *N. Engl. J. Med.* **381**, 668–676 (2019).

14.	The All of Us Research Program Genomics Investigators *et al.* Genomic data in the All of Us Research Program. *Nature* **627**, 340–346 (2024).

15.	Davies, N. M., Dickson, M., Davey Smith, G., Windmeijer, F. & Van Den Berg, G. J. The causal effects of education on adult health, mortality and income: evidence from Mendelian randomization and the raising of the school leaving age. *Int. J. Epidemiol.* **52**, 1878–1886 (2023).

16.	Johnson, S. C. *et al.* The Wisconsin Registry for Alzheimer’s Prevention: A review of findings and current directions. *Alzheimers Dement. Diagn. Assess. Dis. Monit.* **10**, 130–142 (2018).

17.	Ma, Y. *et al.* Comparison of sample characteristics of Wisconsin Alzheimer’s Disease Research Center participants with the Wisconsin state population—An evaluation of the recruitment effort. *Alzheimers Dement. Transl. Res. Clin. Interv.* **11**, e70036 (2025).

18.	Alexander, D. H., Novembre, J. & Lange, K. Fast model-based estimation of ancestry in unrelated individuals. *Genome Res.* **19**, 1655–1664 (2009).

19.	The 1000 Genomes Project Consortium *et al.* A global reference for human genetic variation. *Nature* **526**, 68–74 (2015).

20.	Manichaikul, A. *et al.* Robust relationship inference in genome-wide association studies. *Bioinformatics* **26**, 2867–2873 (2010).
